# Photophysiology data from smooth cordgrass (*Sporobolus alterniflorus*) measured in a North American saltmarsh using pulse amplitude modulated (PAM) fluorescence

**DOI:** 10.1016/j.dib.2025.111458

**Published:** 2025-03-15

**Authors:** Daniel Conrad Ogilvie Thornton

**Affiliations:** Department of Oceanography, 3146 TAMU, Texas A&M University, College Station, 77843, TX, USA

**Keywords:** Electron transport rates, North Inlet-Winyah Bay, Photosynthesis, Photosystem II, Primary production, Quantum yield, Rapid light curves, *Spartina alterniflora*

## Abstract

Coastal saltmarshes play an important role as an interface between terrestrial and marine environments. *Sporobolus alterniflorus* (smooth cordgrass) occurs naturally along the east coast of North America, from Texas to Quebec, where it often forms extensive monospecific stands. *Sporobolus alterniflorus* is highly productive and is often the dominant plant in terms of biomass. This data set presents variable chlorophyll fluorescence measurements made *in situ* from the leaves of *Sporobolus alterniflorus* growing in a tidal saltmarsh ecosystem (North Inlet, South Carolina, United states). Measurements were made using pulse amplitude modulated (PAM) fluorescence. The data include raw measurements of variable fluorescence (*F_t_*) and maximum fluorescence (*F’_m_*) made at 12 different actinic photosynthetic photon flux densities (PPFD). These data were used to calculate the quantum yield of photosystem II *(Φ_PSII_*) and estimate electron transport rates (ETRs). Rapid light curves (RLCs) were fitted to the ETRs to parametrize the relationship between ETR and PPFD in *S. alterniflorus* under different environmental conditions. Measurements were made from *S. alterniflorus* culms growing at different positions on the shore and at different times of the day. These data provide a resource for researchers interested in the photophysiology and photosynthesis of *Sporobolus alterniflorus*, and saltmarsh ecology and management.

Specifications Table

**Table:** 

Subject	Biological sciences
Specific subject area	Marine biology; Plant science: Physiology; Environmental Science: Ecology
Type of data	Table, Raw, Processed (.tab)
Data collection	Data were collected by measuring variable chlorophyll fluorescence of plants *in situ*. Measurements were made using a pulse amplitude modulated (PAM) fluorometer (PAM-210, Heinz Walz GmbH, Germany) in stand-alone operation mode in the field using battery power. Data and supporting notes were recorded in a field notebook. No data were excluded from the analysis, though some data are missing due to logistical issues in the field.
Data source location	Data collected in North Inlet, which lies within the North Inlet-Winyah Bay National Estuarine Research Reserve (NERR), South Carolina, United States. Measurements were made within 300 m of the NERR monitoring station at Oyster Landing (33° 20.977N, 79° 11.331 W).
Data accessibility	Thornton, Daniel, 2024, “Photophysiology of smooth cordgrass (Sporobolus alterniflorus) measured using rapid light curves”, https://doi.org/10.18738/T8/KXKW2S, Texas Data Repository, V2, UNF:6:sBxvRVurZ+I5Oui0FMOL2Q== [fileUNF] Repository name: Texas Data Repository Data identification number: https://doi.org/10.18738/T8/KXKW2S Direct URL to data: https://dataverse.tdl.org/dataverse/Sporobolus_photophysiology/ From the page above, click on the data set to open it to reveal the 8 files it contains. These files can be downloaded to your local drive or viewed within the Texas Data Repository.
Related research article	

## 1 Value of the Data

• Smooth cordgrass (*Sporobolus alterniflorus*) is frequently the dominant intertidal plant in saltmarshes on the east coast of North America. It significantly contributes to ecosystem structure and function as a major primary producer and provides food and habitat for many animal species.
• *Sporobolus alterniflorus* is used in coastal restoration as it can provide significant erosion protection in coastal wetlands.
• There is global interest in *Sporobolus alterniflorus* as it is an invasive species in several locations, including coastal regions of the West Coast of the United States and China.
• This dataset has potential value to researchers interested in the physiology of saltmarsh plants and grasses, plant photophysiology, and estuarine ecosystems.
• These data contribute to the comprehensive and long-term scientific study of North Inlet, which has been studied extensively since North Inlet-Winyah Bay was established as a National Estuarine Research Reserve in 1992.

## 2 Background

*Sporobolus alterniflorus* is widely distributed along the east coast of North America, from Texas to Quebec [1,2]. Variable chlorophyll fluorescence has been used as a tool to study photosynthesis in *S. alterniflorus*; however, the focus of this previous work was to determine variation in productivity over the growing season [1] or how the grass responds to the tidal cycle [3]. There has been little work on light utilization by *S. alterniflorus* and how it responds to short term variations in photosynthetic photon flux density (PPFD), such as those observed on an hourly basis over the course of the day. Here, pulse amplitude modulated (PAM) fluorescence was used to characterise light utilization by *S. alterniflorus* at different positions in the marsh by measuring the quantum yield of PSII (*Φ_PSII_*) over the course of the diurnal light cycle [4].

## 3 Data Description

The raw data are organized into 5 Data sets that correspond to the 5 sets of observations described in the methods (see 4.6 to 4.10). Data from Data set 1, Data set 4, and Data set 5 were processed (see 4.6) to parameterize the rapid light curves (RLCs), producing an addition 3 files of data. The variables and parameters included the data sets are listed in Table 1. Each data set (listed below) is stored in one file in the Texas Data Repository (https://doi.org/10.18738/T8/KXKW2S):

Rapid light curves from a single plant_Data set 1.tab
Quantum yield of photosystem II at different position on leaves_Data set 2.tab
Rapid light curves from 3 leaves on 2 culms_Data set 3.tab
Rapid light curves from multiple culms_June2007_Data set 4.tab
Rapid light curves from multiple culms_August2007_Data set 5.tab
Parameters of rapid light curves from a single plant_Data set 1.tab
Parameters of rapid light curves _June2007_Data set 4.tab
Parameters of rapid light curves _August2007_Data set 5.tab

**Table 1:** List of variables and parameters in the data sets. Variables were measured in the field. Parameters were derived by fitting the photosynthesis-irradiance curve of Jassby and Platt (1976) to the relationship between electron transport rate (ETR) and photosynthetic photon flux density (PPFD).

Variable name	Symbol	Units
Variable fluorescence	*F_t_*	unitless
Maximum fluorescence	*F’_m_*	unitless
Quantum yield of photosystem II	*Φ_PSII_*	unitless
Electron transport rate (ETR)	ETR	μmol e^−^ (m^−2^) s^−1^
Photosynthetic photon flux density (PPFD)	*I*	μmol m^−2^ s^−1^
Air temperature	*T*	°C
**Parameter**		
Alpha	*α*	mol e^−^ (mol photons)^−1^
Maximum electron transport rate	ETR_max_	μmol e^−^ (m^−2^) s^−1^
Saturating photosynthetic photon flux density	*I_k_*	μmol m^−2^ s^−1^

**Fig. 1:**
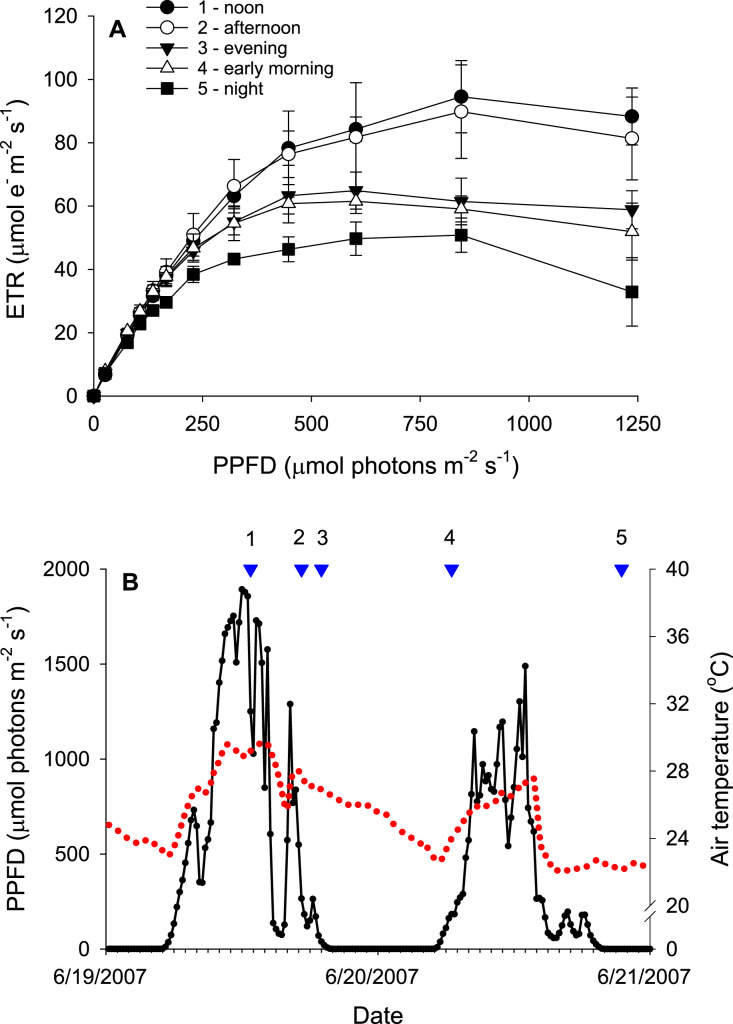
Example plots of rapid light curves (RLCs) and concurrent environmental conditions. **A**. RLCs measured from a single culm of *Sporobolus alterniflorus* several times over a 48 h period. Each data point represents the mean ± standard deviation (n = 3). **B.** Environmental conditions during the RLC measurements. The red dashed line shows the air temperature (°C) and the photosynthetic photon flux density (PPFD) (µmol m^−2^ s^−1^) is shown as black circles. The blue triangles indicate when the RLCs were measured, with the numbers above each triangle indicating the corresponding RLC in **A**.

Fig. 1A provides an example of the data, plotted as a rapid light curve (RLC). Environmental conditions (temperature and PPFD) when the rapid light curve was made are shown in Fig. 1B.

## 4 Experimental Design, Materials and Methods

### 4.1 Field site

The photosynthesis of smooth cordgrass (*Sporobolus alterniflorus*) was measured from culms in North Inlet (South Carolina, USA). North Inlet is a relatively pristine salt marsh (32 km^2^) located within the North Inlet-Winyah Bay National Estuarine Research Reserve (NERR) [5]. All *in situ* measurements were made on *S. alterniflorus* culms in North Inlet within 300 m of the NERR monitoring station at Oyster Landing (33° 20.977N, 79° 11.331 W). North Inlet is a shallow estuary with a semidiurnal tidal pattern (1.5 m mean tidal range) [5,6]. *Sporobolus alterniflorus* is the dominant macrophyte and it grows throughout the salt marsh, except on the high marsh and in the beds of creeks. Field work was conducted in June and August 2007.

### 4.2 Measurement of chlorophyll fluorescence

Chlorophyll fluorescence was measured using a pulse amplitude modulated (PAM) fluorometer (PAM-210, Heinz Walz GmbH, Effeltrich, Germany), which uses LEDs as its light source. The instrument was used in the stand-alone mode of operation in the field under battery power. Measurements were made directly on the measuring head of the instrument, with the adaxial surface of the leaf blade held against the measuring head with a magnetic leaf clip. Measurements were made from the mid-section of each leaf. Defining an individual in a salt marsh is problematic as individual above ground stems of *S. alterniflorus* (i.e. culms) are not genetically distinct individual plants as a single rhizome may produce multiple culms. Furthermore, populations of plants in a salt marsh may be a clonal population of ramets. In this research, distinct above ground structures were regarded as individuals, therefore replicate measurements were taken from replicate culms.

Rapid light curves (described and defined in [7]) were measured using the rapid light curve (RLC) program stored in the PAM-210. After variable fluorescence (*F_t_*) had stabilized (< 5 seconds) the measurements were initiated by applying a single saturating pulse of light (3500 μmol m^−2^ s^−1^) for 1 second at a modulation frequency of 8 KHz. After this initial saturating pulse the measuring light (modulation frequency of 32 Hz; peak wavelength 665 nm with a short-pass filter cutting off wavelengths < 690 nm, resulting in a negligible PPFD of < 0.25 μmol m^−2^ s^−1^ at level 11) was switched to high frequency (8 kHz) for 20 seconds (illuminating the sample with a PPFD of 26 µmol photons m^−2^ s^−1^) and then a second saturating pulse was applied to the sample. Actinic light (8 KHz modulation frequency, peak wavelength at 665 nm) was then applied to the sample for 20 seconds at the level 1 setting on the instrument (illuminating the sample with a total PPFD of 77 µmol photons m^−2^ s^−1^). This was followed by a saturating pulse. Subsequently, actinic light was increased to level 2 and after 20 seconds another saturating pulse was applied. This was repeated to produce a total of 12 measurements over a range of PPFD from 0 to 1237 µmol photons m^−2^ s^−1^. Each measurement consisted of variable fluorescence (*F_t_*) immediately before the saturating pulse and the maximum fluorescence (*F’_m_*) affected by the saturating pulse.

### 4.3 Measurement of Photosynthetic photon flux density

Photosynthetic photon flux density (PPFD) was measured in August using a flat cosine-corrected LiCor (LiCor, Lincoln, Nebraska, USA) underwater sensor (LI-192), which was calibrated for use in air and attached to a meter (250A light meter, LiCor, Lincoln, Nebraska, USA). PPFD data was also obtained from the NERR monitoring station at Oyster Landing (Erik Smith, pers. comm.). These data provided a continuous record of 15 minute averages of PPFD in June and August and were measured using a flat cosine-corrected LiCor sensor (LI-190). The PPFD applied to the sample during the measurement of RLCs was calibrated by placing the center of a LiCor sensor (LI-192) directly on the measuring head of the PAM-210 as if it were a leaf and measuring the PPFD produced by the LEDs in the PAM-210 during a RLC run. A correction factor was applied to account for the fact that the illuminated part of the PAM-210 measuring head was smaller than the area of the LiCor sensor.

### 4.4 Temperature

Air temperature was obtained from the NERR monitoring station at Oyster Landing (downloaded from the Centralized Data Management Office, North Inlet-Winyah Bay NERR; http://cdmo.baruch.sc.edu). The NERR data provided a continuous record of 15 minute averages of air temperature in June and August.

### 4.5 Data processing

The quantum yield of PSII (*Φ_PSII_*) was automatically calculated by the PAM-210 using the measured values of *F_t_* and *F’_m_* using the formula described in [8,9]:

(1) $$\begin{array}{c} \Phi_{\mathrm{PSII}}=\left(F{\text{'}}_{m}-F_{t}\right)/F{\text{'}}_{m} \end{array}$$

During most measurements, the leaves were exposed to *in situ* illumination before measurements were made, as the objective was to determine the photosynthetic response of *Sporobolus alterniflorus* under ambient conditions. Therefore, the commonly measured maximum quantum yield of PSII (*F_v_*/*F_m_*) was not determined, except when the culms had been in the dark for at least 20 minutes, which was the case when the first saturating pulse of a RLC was applied to culms measured at night. The first *Φ_PSII_* of an RLC represents the *Φ_PSII_* under ambient PPFD at the time of measurement.

Electron transport rates (ETR) were calculated using *Φ_PSII_* and the PPFD (*I*) applied to the sample prior to application of the saturating pulse:

(2) $$\begin{array}{c} \text{ETR}=0.5\times \Phi_{\mathrm{PSII}}\times 0.84\times I \end{array}$$

There are two assumptions implicit in the above equation; firstly, that there is an equal distribution of light quanta between photosystems I and II and therefore 50% of absorbed quanta are distributed to PSII (see [1,7,9] for an explanation). Secondly, the proportion of quanta reaching PSII that are absorbed is assumed to be 0.84. This absorptance factor is difficult to measure and species-specific. However, it is often assumed to be 0.84 based on work on work with terrestrial plants [7,10]. However, the ETRs presented should be regarded as relative rather than absolute values.

Curves were fitted to plots of ETR against PPFD using the photosynthesis-irradiance curve of Jassby and Platt (1976) [11]:

(3) $$\begin{array}{c} \text{ETR}=\text{ET}R_{\max}\tanh\left(\alpha I/\text{ET}R_{\max}\right) \end{array}$$

Where ETR was electron transport rate (μmol e^−^ m^−2^ s^−1^), ETR_max_ was the maximum rate of ETR (i.e. the ETR at the plateau of the curve) and α was the initial slope of the curve (mol e^−^ (mol photons)^−1^) and *I* was the PPFD (µmol photons m^−2^ s^−1^). The ratio ETR_max_/α was used to determine the saturating PPFD (*I_k_*). As this simple model does not contain a term for photoinhibition, data from the highest PPFD (1237 µmol photons m^−2^ s^−1^) was not used in the curve fits.

### 4.6 Data set 1: Measurements of RLCs from a single culm in situ

Measurements were made from a single culm (Culm 1) located in the mid marsh in the transition zone between the short and tall ecotypes of *Sporobolus alterniflorus*. Culm 1 was growing next to a boardwalk at 33°20.959N 79°11.564W. Culm 1 was 99 cm tall (from the sediment surface to tip of the youngest leaf) and had 9 healthy leaves. It was relatively tall compared to most of the surrounding culms. Three RLCs were measured from the adaxial surface of 3 different leaves from Culm 1 on each sampling occasion. Measurements were started on 19 June 2007. RLCs were measured on 5 occasions: between 11:45 and 12:00 (noon), at low tide between 17:20 and 17:35 (afternoon), between 19:05 and 19:20 (evening), the next morning on 20 June between 6:30 and 6:45 (early morning), and between 21:30 and 21:45 (night).

### 4.7 Data set 2: Effect of where the measurement is made on a leaf on the quantum yield of PSII

The objective of these measurements were to determine whether position on the leaf has a significant effect on the measurement of *Φ_PSII_*. The youngest fully developed leaf was cut from three culms at 15:20 on 24 June 2007 (33°21.038N, 79°11.530W). The leaves were returned to the laboratory and placed with their adaxial surface facing upwards on tray covered in a damp paper towel. Measurements were started at 16:00. To reduce the time for measurements to be made, a single *Φ_PSII_* was measured rather than full RLCs. The adaxial surface of the leaf was placed on the measuring head of the PAM-210 and exposed to measuring light at level 5 (32 Hz) and actinic light at level 5 (8 KHz). As the measuring light was at a low modulation frequency, it did not apply a significant PPFD to the sample. The actinic light was 200 µmol photons m^−2^ s^−1^. After 1 minute, *F_t_* was recorded and immediately a saturating pulse of light was applied to the sample and *F’_m_* was measured. Measurements were made from the adaxial surface of the proximal end of each leaf (i.e. near where the leaf joined the culm stem), followed by the middle section and the distal section (tip of the leaf). This was repeated to produce three measurements in each section of the leaf. Replicate measurements were not made from exactly the same spot in each section. Measurements took 30 minutes.

Measurements were made to compare *Φ_PSII_* on the adaxial and abaxial surfaces of the leaves. The two surfaces of the leaves would have been exposed to different light regimes while lying on the tray in the laboratory. Therefore, leaves were placed in the dark for 40 minutes prior to measurement. To prevent the leaves from drying out, they were placed in a Ziploc bag (S.C. Johnson & Son, Inc.) with a damp paper towel.

### 4.8 Data set 3: In situ RLCs from multiple leaves from two culms

The objective of these measurements was to determine whether there was as significant difference between RLCs measured in different leaves from the same culm or between culms. Measurements were made on two culms (from Site 3; 33°21.100N, 79°11.489W) *in situ* and the leaves were not removed from the culms. RLCs were measured on two leaves from each culm on 28 June 2007 between 10:20 and 11:15. Three RLCs were measured from the middle section of each leaf. The point of measurement was moved for each RLC so that measurements were not made in exactly the same place twice.

### 4.9 Data set 4: In situ comparison between culms at two sites (June)

Four culms were arbitrarily selected in the upper edge (Site 1) of the *Spartina alterniflora* zone (33°21.059N, 79°11.511W). An additional four culms were selected in the lower edge (Site 3) of the *S. alterniflorus* zone near the banks of Crab Haul Creek (33°21.100N, 79°11.489W). The upper intertidal *S. alterniflora* were inundated with seawater for less time than the culms on the low shore. Culms on the low shore were the tall ecotype of *S. alterniflorus*, whereas those on the upper shore were intermediate between the short and tall ecotypes. A single RLC was measured from one attached leaf on each culm on each sampling occasion. Measurements were made mid-afternoon (14:30 to 15:05) on 24 June, evening (19:55 to 20:35) on 25 June, early morning (6:50 to 7:25) on 26 June, mid-morning (10:40 to 11:30) on 26 June, and late afternoon (16:40 to 17:25) on 26 June.

### 4.10 Data set 5: In situ comparison between culms at two sites (August)

Four *Sporobolus alterniflorus* culms were selected at Site 3 by the bank of Crab Haul Creek. An additional four culms were selected in the short *S. alterniflorus* zone between Site 3 and Site 1. This site (33°21.083N, 79°11.502W) was called Site 2 and enabled comparison between the short and tall ecotypes of *S. alterniflorus*. A single RLC was measured from one attached leaf on each culm on each sampling occasion. Measurements were made from Site 3 in the afternoon (15:00 to 15:30) on 21 August, evening (18:55 to 19:25) on 21 August, morning (9:10 to 9:40) on 23 August, afternoon (15:40 to 16:10) on 24 August, and late afternoon (17:20 to 17:45) on 24 August. Measurements were made from Site 2 on the evening 21 August (18:30 to 18:55) and afternoon 24 August (15:15 to 15:35).

## Limitations

A small number of measurements are missing from the data due to logistical challenges encountered during fieldwork.

## Ethics Statement

The author has read and followed the ethical requirements for *Data in Brief*. The methods and data associated with this paper did not involve human subjects, animal experiments, or any data collected from social media platforms.

## CRedit Author Statement

**Daniel C. O. Thornton** was responsible for all aspects of this work (conceptualization, methodology, data collection, analysis of the data, writing and editing).

## Data Availability

Texas Data RepositoryPhotophysiology of smooth cordgrass (Sporobolus alterniflorus) measured using rapid light curves (Original data).
